# TIMP3 involvement and potentiality in the diagnosis, prognosis and treatment of diabetic nephropathy

**DOI:** 10.1007/s00592-021-01766-y

**Published:** 2021-06-28

**Authors:** Viviana Casagrande, Massimo Federici, Rossella Menghini

**Affiliations:** 1 Departments of Systems Medicine, University of Rome “Tor Vergata”, Rome, Italy; 2 Center for Atherosclerosis, Department of Medical Sciences, Policlinico Tor Vergata University, Rome, Italy

**Keywords:** Diabetic nephropathy, TIMP3, Noncoding RNA

## Abstract

Diabetic kidney disease, one of the most severe complications associated with diabetes, is characterized by albuminuria, glomerulosclerosis and progressive loss of renal function. Loss of TIMP3, an Extracellular matrix-bound protein, is a hallmark of diabetic nephropathy in human and mouse models, suggesting its pivotal role in renal diseases associated to diabetes. There is currently no specific therapy for diabetic nephropathy, and the ability to restore high TIMP3 activity specifically in the kidney may represent a potential therapeutic strategy for the amelioration of renal injury under conditions in which its reduction is directly related to the disease. Increasing evidence shows that diabetic nephropathy is also regulated by epigenetic mechanisms, including noncoding RNA. This review recapitulates the pathological, diagnostic and therapeutic potential roles of TIMP3 and the noncoding RNA (microRNA, long noncoding RNA) related to its expression, in the progression of diabetic nephropathy.

## Introduction

Diabetic nephropathy (DN) is a major complication of type I and II diabetes and the main cause of end stage renal disease (ESRD) [1]. The clinical and pathological hallmarks of DN are progressive albuminuria, followed by a gradual decline in glomerular filtration rate (GFR) and podocyte loss. Morphologically, DN is characterized by changes in glomerular basement membrane thickness and content as well as mesangial expansion finally leading to tubulointerstitial fibrosis [2]. Over the past few years, many studies have contributed to the general understanding of the plethora of signaling pathways abnormalities that have a pathogenic role in DN [3]. Hodgin and colleagues performed a comparison of glomerular gene expression in diabetic patients and in three mouse models of diabetes (streptozotocin (STZ)-treated, db/db and eNOS^−/−^db/db mice) [4]. This analysis led to the identification of three cross-species glomerular transcriptional networks shared between humans and each mouse model. Each network was characterized by several gene nodes, and nodes common to all networks represented established pathogenic mechanisms of DN in human and mice. Interestingly, novel pathways not previously associated with DN were also highlighted; among them, two members of the Tissue Inhibitor of Metalloproteinase (TIMP) family, TIMP2 and TIMP3, emerged as candidate genes both in human and in two out of three mouse models of diabetes analyzed (STZ and db/db mice). TIMP3 is an Extracellular matrix (ECM)-bound protein that is widely expressed in humans and mice. By virtue of its modulatory activity, it plays an important function in regulating matrix composition, thereby affecting a wide range of physiological processes such as cell growth and migration, angiogenesis and apoptosis.

## TIMP3 and diabetic nephropathy

TIMP3 is the most highly expressed TIMP in the kidney [5] and has a broad protease inhibition profile; its loss associates with age-related renal fibrosis and tubulointerstitial fibrosis [6, 7], which are important prognostic markers in wide variety of kidney diseases. We and others have demonstrated that loss of TIMP3 contributes to the onset and progression of diabetic kidney disease (DKD) in mouse models of diabetes [8, 9]. We have shown that TIMP3 expression was decreased in the kidney of STZ treated-mice, a well-established model of hyperglycemia and glucotoxicity, reproducing Type 1 diabetic disease [8]. Timp3^−/−^ diabetic kidneys showed a higher degree of inflammation and podocyte dysfunction compared to WT diabetic control, indicating that loss of TIMP3 is detrimental to the progression of DKD. In addition, a study from Basu et al. [9] showed that in Timp3^−/−^ Akita diabetic mice, loss of TIMP3 worsened diabetic renal injury, as demonstrated by mesangial expansion and increased microalbuminuria. Exacerbation of diabetic renal damage by deletion of TIMP3 was also associated with increased NADPH activity and oxidative stress, as well as upregulation of inflammatory and fibrotic markers [9]. Akita mice developed cardiac diastolic dysfunction, however TIMP3 deficiency did not aggravate this diabetic cardiomyopathy, unveiling a key and organ-specific role for TIMP3 in DN [10].

Previous studies have focused on the involvement of TIMP3 in human kidney pathology [6, 7]. In a family-based genetic study, 115 candidate genes for linkage and association with diabetic nephropathy were analyzed using the transmission/disequilibrium test [11]. Among them, Timp3 polymorphisms showed a significant association with diabetic nephropathy, suggesting that allelic variations of this gene may contribute to the risk of developing the disease and pointing at Timp3 as a susceptibility gene for DKD. Moreover, a transcriptome analysis of human kidney biopsies, showed that TIMP3 was specifically down-regulated in glomeruli but not in tubuli of diabetic subjects, compared to healthy controls [12], suggesting for TIMP3 different roles in these distinct compartments. In kidney biopsies from diabetic patients, we also founded a significant decrease of TIMP3 expression especially in diabetic glomeruli compared to the controls [8]. Evidence from renal biopsies has shown that macrophage accumulation in diabetic kidneys predicts declining renal function, suggesting a pathogenic role for these cells in diabetic nephropathy. Further evidence from animal models has shown that macrophages are the major immune cells infiltrating the kidney in type 1 and type 2 diabetes, and that they contribute to the development of renal injury and sclerosis [13]. We have generated a mouse model with cell-targeted overexpression of TIMP3 in myeloid cells (MacT3), which results in overexpression of TIMP3 directly at the sites in which monocytes/macrophages are gradually recruited during disease progression. We have already shown that, MacT3 mice are protected from inflammation and related metabolic disorders during obesity [14] and from the progression of vascular damage associated with atherosclerosis [15]. Next, we treated MacT3 mice with the pancreatic islet cell toxin STZ in order to test the protective effect of the overexpression of TIMP3, directly in the kidney, during the progression of diabetes. Results indicated that MacT3 mice after 12 weeks from the induction of diabetes by a low-dose STZ protocol treatment, showed a reduction in renal lesions, albuminuria, inflammation and fibrosis and prevention of the loss of podocytes [16], suggesting that TIMP3 may halt or slow the progression of diabetic kidney complications, thus representing a valid approach to characterize the pathogenesis of DN and to develop new avenues to diagnose and treat this disorder. Consistently with our results, conditional ablation of TNF-α, a crucial ADAM17 substrate, in macrophages in Akita mice conferred kidney protection after 12 weeks of STZ-induced diabetes [17].

## TIMP3 targets and diabetic nephropathy

TIMP3 is a known physiological inhibitor of ADAM17, a metalloprotease responsible for shedding of several ligands; among these, HB-EGF and TGF-β are involved in the pathogenesis of chronic kidney disease and glomerulonephritis [18, 19]. ADAM17 also participates in the generation of the transcriptionally active form of Notch, which is important for glomerular and proximal tubules development as well as regulation of podocytes dysfunction [20, 21]. Interestingly, elevated plasma concentration of two ADAM17 substrates such as TNFR1 and TNFR2 have been recently found to predict Stage 3 Chronic Kidney Disease and End Stage Renal Disease in patients with type 1 and type 2 diabetes, respectively, even in the absence of proteinuria [22, 23]. ADAM17 expression and activity were found increased in the kidney cortex of OVE26 mice with type 1 diabetes and in renal cells exposed to high glucose concentrations; inhibition of this metalloprotease led to a decreased deposition of matrix proteins such as collagen IV and fibronectin, along with decreased Nox4 expression and NADPH oxidase activity [24]. Several groups demonstrated a role for ADAM17 in mediating the profibrotic effect of angiotensin II (AngII). A cross-talk between AngII and EGFR has been shown to play a pivotal role in stimulating the development of renal lesions [19]; chronic infusion of AngII in mice resulted in glomerulosclerosis and interstitial fibrosis, while transgenic mice for a dominant negative isoform of EGFR or TGF-β^−/−^ mice were protected from these lesions. Importantly, AngII-induced renal lesions were reduced in WT mice administered a pharmacological inhibitor of ADAM17. AngII also causes redistribution of this metalloprotease to the apical membrane of renal tubules [19]. ADAM17 has also been involved in the ectodomain shedding of angiotensin converting enzyme (ACE) 2, a new enzyme within the renin angiotensin system (RAS) [25, 26]. Recently, it has been demonstrated that shedding of renal ACE2 into urine is increased in db/db diabetic mice similar to the expression of ADAM17 in the kidney [27]: due to the action of ADAM17, proteolytically active form of ACE2 are shed from the kidney into urine of db/db mice, and this loss of the renoprotective enzyme ACE2 could contribute to kidney damage [27]. Beside ROS and AngII, several stimuli can increase ADAM17 activity in a tissue or cell-specific manner [28–30]. Hyperglycemia, hyperinsulinemia, free fatty acids and endotoxin can all induce ADAM17 activation in different cell lines, as well as in mouse metabolic tissues [28, 30]. The involvement of TIMP3/ADAM17 pathway in the control of glucose homeostasis and adipose and vascular inflammation in patients with obesity-related T2DM and atherosclerosis has been already shown [31, 32]. Moreover, low-dose Pioglitazone (PIO), a peroxisome proliferator-activated receptor (PPAR)γ agonist, reduced ADAM17 enzymatic activity in human skeletal muscle, and that these effects were associated with an improvement in glyco-metabolic control and inflammatory state in type 2 diabetes [33]. We previously tested the effect of selective genetic inhibition of ADAM17 in hepatocyte or myeloid cells on glucose metabolism and inflammatory status, observing a protection from diet induced insulin resistance and hepatic inflammation [34]. Moreover, others have shown that selective genetic inhibition of ADAM17 in specific cell types could be beneficial for treatment of several pathologies, including proliferative retinopathies, rheumatoid arthritis and cancer [35, 36]. In particular, KO of ADAM17 in proximal tubule (Slc34a1-Cre) were significantly protected against inflammation and fibrosis after kidney injury (including ischemia and ureteral obstruction) [37]. It is now recognized that interference with podocyte specific disease pathways can modulate glomerular function and influence severity and progression of glomerular disease. We recently conditionally inactivated ADAM17 in podocytes and then determined how specific lack of ADAM17 affects the progression of kidney disease induced by STZ treatment [16]. Our findings indicate that conditional deletion of ADAM17 in podocytes improves albuminuria and ameliorates progression of DN, protecting podocytes which have been recognized as critical regulators of glomerular injury.

Matrix metalloproteinases (MMPs) were previously known to be anti-fibrotic for their ability to degrade and remodel extracellular matrix proteins. However, recently MMP-2 and MMP-9, whose activity is regulated by TIMP3, were found to be able to induce epithelial–mesenchymal transition of tubular cells as well as endothelial–mesenchymal transition, both important mechanisms causing kidney fibrosis in diabetic nephropathy [38–40]. Thus, TIMP3 may play a role in maintaining kidney homeostasis. Overall, previous reports and our data indicate that rescuing TIMP3 functions may represent a new therapeutic approach to block the progression of diabetic nephropathy.

## Role of noncoding RNAs regulating TIMP3 in diabetic nephropathy

**Fig. 1:**
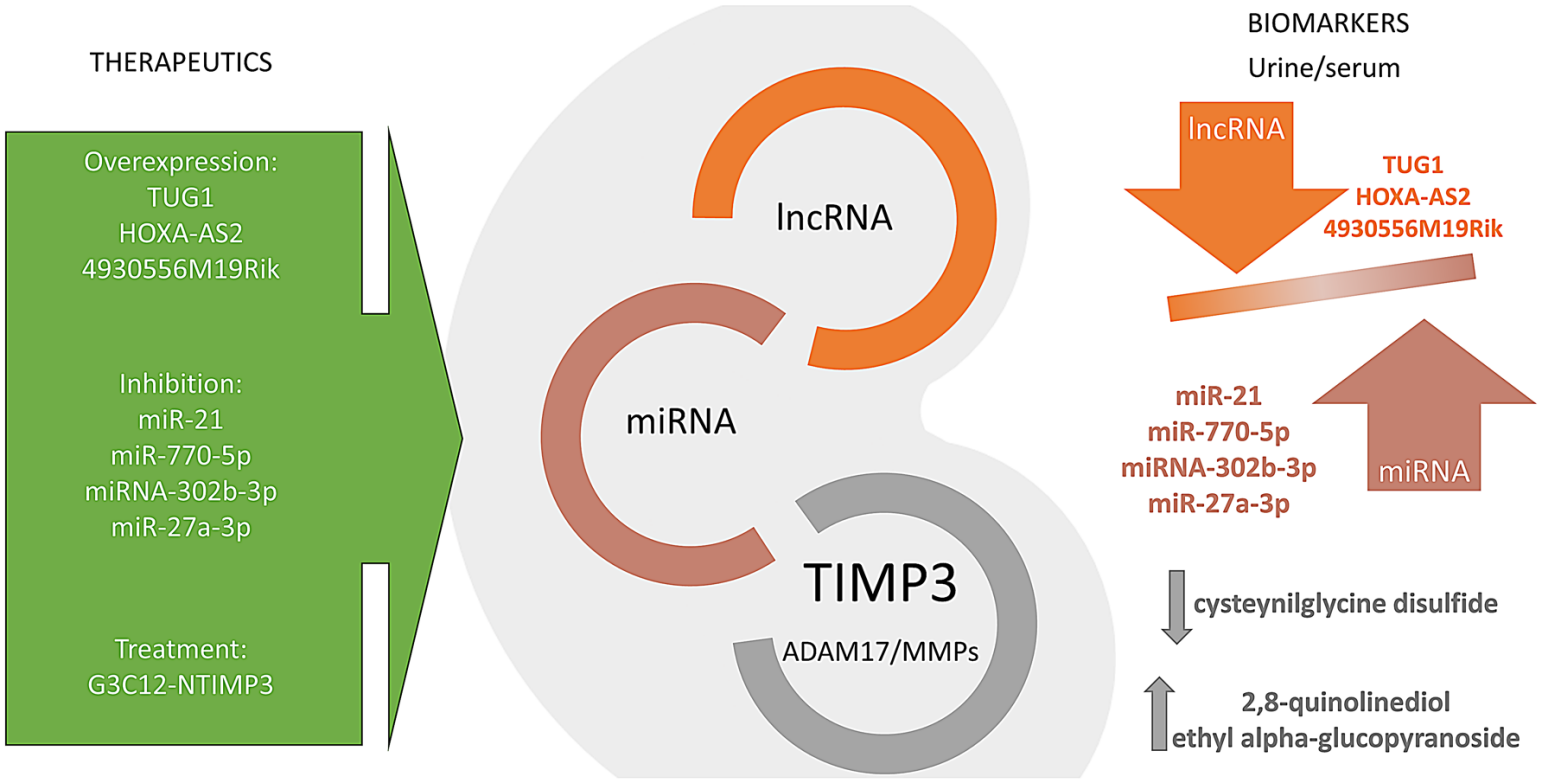
lncRNA–miRNA–TIMP3 axes: lncRNAs acting as miRNAs sponges enhanced the expression of TIMP3, whose reduction represents a hallmark of diabetic nephropathy. These specific lncRNAs and miRNAs, combined with TIMP3 expression, may be considered as biomarkers and therapeutic targets for diabetic kidney disease

Noncoding RNAs (ncRNAs) are a class of RNAs produced by genome transcription with no or low coding potential. NcRNAs participate in the pathogenesis of several diseases, including DN, by regulating different pathological processes [41, 42]. The main classes of functional ncRNAs include microRNA (miRNA), long noncoding RNA (lncRNA) and circular RNA (circRNA). MicroRNAs (miRNAs) are short noncoding, evolutionarily conserved RNAs that post-transcriptionally regulate gene expression by binding the 3′ untranslated region (3′ UTR) of mRNA. More recently, the involvement of miRNAs in renal pathophysiology has gained wide attentions, especially in DN where modulation of miRNAs may act in a tissue or cell-specific manner [43]. LncRNAs are a novel type of noncoding RNAs (longer than 200 nucleotides) without protein-encoding abilities. LncRNAs are widely involved in various life activities through epigenetics and transcription regulation. Some studies have demonstrated the critical impacts of certain lncRNAs on the incidence and progression of DN [44]. Recently, a new regulatory loop “lncRNA–miRNA–mRNA” has been proposed: lncRNAs acting as miRNAs sponges reduced their effects on target mRNAs, thereby enhancing the expression of these genes [45]. A similar role is emerging for circRNAs, a class of newly identified ncRNAs without either polyadenylated tails in 3′ ends or the cap structure at 5′ ends [41]. Given the emerging role of TIMP3 deficiency in DN in mice and human, several studies have been focused on specific ncRNAs that could affect TIMP3 expression in the context of diabetic renal disease. In particular, TIMP3 expression was inversely correlated with miR-21 levels in glomeruli of patients with DN, and different studies have found that miR-21 could be implicated in TIMP3 regulation [46]. The down-regulation of miR-21 weakened kidney injury and pro-inflammatory responses in STZ-induced DN rats [46, 47]. Moreover, miR-21 overexpression promoted inflammatory responses and cell apoptosis by targeting TIMP3 in HG-treated podocytes, whereas TIMP3 overexpression suppressed these actions. Consistently, miR-21 inhibitor replaced TIMP3 expression in HG-treated podocytes [47], demonstrating that miR-21 hampered the development and progression of DN both in vivo and in vitro by targeting TIMP3, effects that may imply a therapeutic approach to slow DN. Additionally, in podocytes TIMP3 was negatively regulated by miR-770-5p whose expression was increased in DN and miR-770-5p inhibitor improved HG induced inflammation and apoptosis avoiding TIMP3 reduction [48]. Recently, modulation of different lncRNA–miRNA–mRNA axes has been proposed for the regulation of TIMP3 in DN (Fig. 1). First, lncRNA TUG1, that played an important role in DN progress, was found to act as the sponge for miR-21, that in turn represents a crucial regulator of TIMP3 [49]. Overexpression of lncRNA TUG1 resulted in down-regulation of miR-21, and upregulation of TIMP3 expression in high glucose-stimulated NRK-52E cells and in DN mice; these effects inhibited cell fibrosis through the attenuation of renal fibrosis marker genes [50]. HOXA-AS2, a lncRNA known to exert a wide range of regulatory functions, was found reduced in serum and kidney from diabetic rat [51]. In vivo overexpression of HOXA-AS2 reduced kidney injuries, serum levels of IL-1β, TNF-α, creatinine, BUN and blood glucose; similarly, in HG-treated podocytes HOXA-AS2 reduced the expression of miRNA-302b-3p damping the down-regulation of TIMP, leading to the reduction of inflammatory response and apoptosis [51]. The 4930556M19Rik/miR-27a-3p/TIMP3 axis is also involved in DN. In fact, 4930556M19Rik was significantly decreased in HG-stimulated podocytes and 4930556M19Rik overexpression alleviated HG induced apoptosis, fibrosis and inflammatory response by downregulating miR-27a-3p and upregulating TIMP3 [52]. Overall, although the role of ncRNAs in the pathogenesis of DN has not been fully elucidated, emerging evidence shows the ncRNAs potential in the control of DN progression through the modulation of TIMP3 expression.

## Potential of TIMP3 as therapeutic target and biomarker in diabetic nephropathy

Recent evidence in pre-clinical studies suggests that TIMP3-based therapy may have broad clinical potential in cancer, inflammatory or cardiovascular diseases [53]. However, the administration of MMP or ADAM17 inhibitors in experimental models to obtain clinical translational results has been a challenge. To this end, several strategies to increase TIMP3 delivery in specific cells have been explored, including incorporation into MMP-sensitive hydrogel, fusion with the latency‐associated peptide (LAP) or cell-based gene transfer [54–56]. Recently, to evaluate the contribution of kidney TIMP3 overexpression to the onset of DN, we created, by chemical synthesis, a new peptide, derived from the fusion of the N-terminal domain of human TIMP3 protein with G3-C12 galectin-3 targeting peptide [16]. Full-length TIMP-3 is difficult to refold from inclusion bodies, and previous papers on TIMP-3 demonstrated that the refolded N-TIMP-3 domain conserved the inhibitor activity against metalloproteinases and ADAM17 [57]. Therefore, we focused on the engineering of this portion of TIMP3 protein. To direct N-TIMP3 derived peptides into the kidney, we conjugated them with a galectin-3 receptor targeting peptide which proved to specifically accumulates in the kidneys after injection and has been already used successfully in conjugation with the ACE inhibitor captopril [58]. The specific binding of G3-C12 to galectin-3 would increase the internalization of large amounts of drug via active endocytosis [59]. Galectin-3 expression increased in diabetic kidney and closely correlated with the regression rate of renal function, therefore representing a favorable target for our system [60]. This approach has been preferred to the use of adenoviral-mediated overexpression of TIMP3 because adenoviruses failed to reach the kidney at the sufficient level requested to modulate the expression of the target that they are carrying to be encoded [61]. The renal delivery of peptides ensures an enzymatic inhibition specifically in the kidney, in the absence of actions of the drug elsewhere in the body. In addition, this may increase the therapeutic effectiveness by allowing higher renal drug concentration. Our in vivo study provides evidence that the treatment with G3C12-NTIMP3 peptide may delay development of nephropathy in diabetic mice, as indicated by reduction of albuminuria, inflammation, fibrosis and glomerular damage [16].

Other powerful strategies aimed to reduce the diabetes dependent detrimental effects on kidney, applied different technologies for the delivery of synthetic ncRNA or inhibitors, to interfere with specific signaling pathways. Interestingly, miR-21 antagonists have shown great potential in the treatment of diabetic complications, including nephropathy. In fact, both in vivo and in vitro, these antagonists are able to reduce albuminuria, renal fibrosis and podocyte damage, in line with results that we obtained with G3C12-NTIMP3 treatment [62–64].

At present, the clinical diagnosis of DN mainly depends on the elevated urinary albumin excretion and reduced GFR in the absence of other primary causes of kidney damage, however none of these measures can accurately indicate the severity and type of injury induced by hyperglycemia and renal biopsy is still the gold standard to diagnose DN. For patients at risk for progressing to DN, early diagnosis and targeted interventions are hindered by the lack of sensitive and accurate tools. Moreover, clinical treatment of DN remains a challenge due to its complex etiology. A growing number of studies have focused on the biomarkers for early diagnosis of DN [65]. Urinary exosomes, plasma/serum or urinary level of different microRNAs, proteins or metabolites are perceived as a potential novel way of detecting DN during its early stages. Serum TIMP3 levels were significantly associated with different inflammatory, cardiovascular o cancer diseases [53]. It is intriguing to speculate that the higher expression of miR-21 in the serum samples of DN patients, that closely reflects expression of miR-21 in the kidney, may represent a new biomarker for TIMP3 levels in kidney tissue in the context of DN. Metabolomics tools have shown great promise in development of diagnostic and prognostic biomarkers as well as in advancing our understanding of the molecular mechanisms underlying the pathology of DN [66]. Metabolomics approach can provide insight into the entire metabolism process and identify disparities in related metabolic pathways. Metabolites could be applied to diagnose and monitor disease progression by evaluating their content variations in response to different treatments [67]. Recently, to identify early specific markers exploitable to clinical diagnosis of DN, we analyzed sera metabolic profile of DN mice treated with G3C12-NTIMP3 peptide, compared to DN untreated mice. We found 7 metabolites specifically associated with peptide treatment. In particular, cysteynilglycine disulfide was found down-regulated and 2,8-quinolinediol and ethyl alpha-glucopyranoside were upregulated in response to the treatment. While further studies are needed to confirm the relevance of these results, it is intriguing to speculate that this specific metabolic signature might be explored as a companion biomarkers for TIMP3-based treatment of diabetic nephropathy.

## Conclusions

Several recent evidences point to TIMP3 as a potential biomarker or therapeutic target for kidney diabetic disease. Given the multiplicity of TIMP3 actions it is plausible that is more appropriate to rescue its level in vivo to obtain a therapeutic effect rather than trying to inhibit all the enzymes or receptors that are overactivated in its absence. It is now demonstrated that several miRNAs or lncRNAs are potential inhibitors of TIMP3 expression. These miRNAs/lncRNAs may be considered as biomarkers but since each of them has multiple targets pointing on their direct inhibition or overactivation may lead to undesirable effects. Therefore, engineering TIMP3 protein in order to be administered might be the most direct approach to rescue its deficiency in the diabetic kidney.

## References

[1] Molitch ME, DeFronzo RA, Franz MJ, Keane WF, Mogensen CE, Parving HH (2003). Diabetic nephropathy. Diabetes Care.

[2] Lane PH, Steffes MW, Mauer SM (1990). Renal histologic changes in diabetes mellitus. Semin Nephrol.

[3] Brosius FC, Khoury CC, Buller CL, Chen S (2010). Abnormalities in signaling pathways in diabetic nephropathy. Expert Rev Endocrinol Metab.

[4] Hodgin JB, Nair V, Zhang H (2013). Identification of cross-species shared transcriptional networks of diabetic nephropathy in human and mouse glomeruli. Diabetes.

[5] Catania JM, Chen G, Parrish AR (2007). Role of matrix metalloproteinases in renal pathophysiologies. Am J Physiol Renal Physiol.

[6] Kawamoto H, Yasuda O, Suzuki T (2006). Tissue inhibitor of metalloproteinase-3 plays important roles in the kidney following unilateral ureteral obstruction. Hypertens Res.

[7] Kassiri Z, Oudit GY, Kandalam V (2009). Loss of TIMP3 enhances interstitial nephritis and fibrosis. J Am Soc Nephrol.

[8] Fiorentino L, Cavalera M, Menini S (2013). Loss of TIMP3 underlies diabetic nephropathy via FoxO1/STAT1 interplay. EMBO Mol Med.

[9] Basu R, Lee J, Wang Z (2012). Loss of TIMP3 selectively exacerbates diabetic nephropathy. Am J Physiol Renal Physiol.

[10] Basu R, Oudit GY, Wang X (2009). Type 1 diabetic cardiomyopathy in the Akita (Ins2WT/C96Y) mouse model is characterized by lipotoxicity and diastolic dysfunction with preserved systolic function. Am J Physiol Heart Circ Physiol.

[11] Ewens KG, George RA, Sharma K, Ziyadeh FN, Spielman RS (2005). Assessment of 115 candidate genes for diabetic nephropathy by transmission/disequilibrium test. Diabetes.

[12] Woroniecka KI, Park AS, Mohtat D, Thomas DB, Pullman JM, Susztak K (2011). Transcriptome analysis of human diabetic kidney disease. Diabetes.

[13] Tesch GH (2010). Macrophages and diabetic nephropathy. Semin Nephrol.

[14] Menghini R, Casagrande V, Menini S (2012). TIMP3 overexpression in macrophages protects from insulin resistance, adipose inflammation, and nonalcoholic fatty liver disease in mice. Diabetes.

[15] Casagrande V, Menghini R, Menini S (2012). Overexpression of tissue inhibitor of metalloproteinase 3 in macrophages reduces atherosclerosis in low-density lipoprotein receptor knockout mice. Arterioscler Thromb Vasc Biol.

[16] Casagrande V, Iuliani G, Menini S, Pugliese G, Federici M, Menghini R (2021). Restoration of renal TIMP3 levels via genetics and pharmacological approach prevents experimental diabetic nephropathy. Clin Transl Med.

[17] Awad AS, You H, Gao T (2015). Macrophage-derived tumor necrosis factor-α mediates diabetic renal injury. Kidney Int.

[18] Bollee G, Flamant M, Schordan S (2011). Epidermal growth factor receptor promotes glomerular injury and renal failure in rapidly progressive crescentic glomerulonephritis. Nat Med.

[19] Lautrette A, Li S, Alili R (2005). Angiotensin II and EGF receptor cross-talk in chronic kidney diseases: a new therapeutic approach. Nat Med.

[20] Murthy A, Shao YW, Narala SR, Molyneux SD, Zuniga-Pflucker JC, Khokha R (2012). Notch activation by the metalloproteinase ADAM17 regulates myeloproliferation and atopic barrier immunity by suppressing epithelial cytokine synthesis. Immunity.

[21] Niranjan T, Bielesz B, Gruenwald A (2008). The Notch pathway in podocytes plays a role in the development of glomerular disease. Nat Med.

[22] Niewczas MA, Gohda T, Skupien J (2012). Circulating TNF receptors 1 and 2 predict ESRD in type 2 diabetes. J Am Soc Nephrol.

[23] Gohda T, Niewczas MA, Ficociello LH (2012). Circulating TNF receptors 1 and 2 predict stage 3 CKD in type 1 diabetes. J Am Soc Nephrol.

[24] Ford BM, Eid AA, Gooz M, Barnes JL, Gorin YC, Abboud HE (2013). ADAM17 mediates Nox4 expression and NADPH oxidase activity in the kidney cortex of OVE26 mice. Am J Physiol Renal Physiol.

[25] Lambert DW, Yarski M, Warner FJ (2005). Tumor necrosis factor-alpha convertase (ADAM17) mediates regulated ectodomain shedding of the severe-acute respiratory syndrome-coronavirus (SARS-CoV) receptor, angiotensin-converting enzyme-2 (ACE2). J Biol Chem.

[26] Jia HP, Look DC, Tan P (2009). Ectodomain shedding of angiotensin converting enzyme 2 in human airway epithelia. Am J Physiol Lung Cell Mol Physiol.

[27] Chodavarapu H, Grobe N, Somineni HK, Salem ES, Madhu M, Elased KM (2013). Rosiglitazone treatment of type 2 diabetic db/db mice attenuates urinary albumin and angiotensin converting enzyme 2 excretion. PLoS ONE.

[28] Fiorentino L, Vivanti A, Cavalera M (2010). Increased tumor necrosis factor alpha-converting enzyme activity induces insulin resistance and hepatosteatosis in mice. Hepatology.

[29] Chen CD, Podvin S, Gillespie E, Leeman SE, Abraham CR (2007). Insulin stimulates the cleavage and release of the extracellular domain of Klotho by ADAM10 and ADAM17. Proc Natl Acad Sci U S A.

[30] Menghini R, Fiorentino L, Casagrande V, Lauro R, Federici M (2013). The role of ADAM17 in metabolic inflammation. Atherosclerosis.

[31] Monroy A, Kamath S, Chavez AO (2009). Impaired regulation of the TNF-alpha converting enzyme/tissue inhibitor of metalloproteinase 3 proteolytic system in skeletal muscle of obese type 2 diabetic patients: a new mechanism of insulin resistance in humans. Diabetologia.

[32] Cardellini M, Menghini R, Martelli E (2009). TIMP3 is reduced in atherosclerotic plaques from subjects with type 2 diabetes and increased by SirT1. Diabetes.

[33] Tripathy D, Daniele G, Fiorentino TV (2013). Pioglitazone improves glucose metabolism and modulates skeletal muscle TIMP-3-TACE dyad in type 2 diabetes mellitus: a randomised, double-blind, placebo-controlled, mechanistic study. Diabetologia.

[34] Casagrande V, Mauriello A, Bischetti S, Mavilio M, Federici M, Menghini R (2017). Hepatocyte specific TIMP3 expression prevents diet dependent fatty liver disease and hepatocellular carcinoma. Sci Rep.

[35] Weskamp G, Mendelson K, Swendeman S (2010). Pathological neovascularization is reduced by inactivation of ADAM17 in endothelial cells but not in pericytes. Circ Res.

[36] Maretzky T, Zhou W, Huang XY, Blobel CP (2011). A transforming Src mutant increases the bioavailability of EGFR ligands via stimulation of the cell-surface metalloproteinase ADAM17. Oncogene.

[37] Kefaloyianni E, Muthu ML, Kaeppler J (2016). ADAM17 substrate release in proximal tubule drives kidney fibrosis. JCI Insight.

[38] Zhao H, Dong Y, Tian X (2013). Matrix metalloproteinases contribute to kidney fibrosis in chronic kidney diseases. World J Nephrol.

[39] Zhao Y, Qiao X, Wang L (2016). Matrix metalloproteinase 9 induces endothelial-mesenchymal transition via Notch activation in human kidney glomerular endothelial cells. BMC Cell Biol.

[40] Matsui F, Babitz SA, Rhee A, Hile KL, Zhang H, Meldrum KK (2017). Mesenchymal stem cells protect against obstruction-induced renal fibrosis by decreasing STAT3 activation and STAT3-dependent MMP-9 production. Am J Physiol Renal Physiol.

[41] Ren H, Wang Q (2021). Non-coding RNA and diabetic kidney disease. DNA Cell Biol.

[42] Gu YY, Lu FH, Huang XR (2021). Non-coding RNAs as biomarkers and therapeutic targets for diabetic kidney disease. Front Pharmacol.

[43] Bhatt K, Kato M, Natarajan R (2016). Mini-review: emerging roles of microRNAs in the pathophysiology of renal diseases. Am J Physiol Renal Physiol.

[44] Lu Z, Liu N, Wang F (2017). Epigenetic regulations in diabetic nephropathy. J Diabetes Res.

[45] Wei C, Luo T, Zou S (2017). Differentially expressed lncRNAs and miRNAs with associated ceRNA networks in aged mice with post operative cognitive dysfunction. Oncotarget.

[46] Lai JY, Luo J, O'Connor C (2015). MicroRNA-21 in glomerular injury. J Am Soc Nephrol.

[47] Chen X, Zhao L, Xing Y, Lin B (2018). Down-regulation of microRNA-21 reduces inflammation and podocyte apoptosis in diabetic nephropathy by relieving the repression of TIMP3 expression. Biomed Pharmacother.

[48] Wang L, Li H (2020). MiR-770-5p facilitates podocyte apoptosis and inflammation in diabetic nephropathy by targeting TIMP3. Biosci Rep.

[49] Arun K, Arunkumar G, Bennet D, Chandramohan SM, Murugan AK, Munirajan AK (2018). Comprehensive analysis of aberrantly expressed lncRNAs and construction of ceRNA network in gastric cancer. Oncotarget.

[50] Wang F, Gao X, Zhang R, Zhao P, Sun Y, Li C (2019). LncRNA TUG1 ameliorates diabetic nephropathy by inhibiting miR-21 to promote TIMP3-expression. Int J Clin Exp Pathol.

[51] Li X, Yu HM (2020). Overexpression of HOXA-AS2 inhibits inflammation and apoptosis in podocytes via sponging miRNA-302b-3p to upregulate TIMP3. Eur Rev Med Pharmacol Sci.

[52] Fan H, Zhang W (2020). Overexpression of Linc 4930556M19Rik suppresses high glucose-triggered podocyte apoptosis, fibrosis and inflammation via the miR-27a-3p/metalloproteinase 3 (TIMP3) axis in diabetic nephropathy. Med Sci Monit.

[53] Fan D, Kassiri Z (2020). Biology of tissue inhibitor of metalloproteinase 3 (TIMP3), and its therapeutic implications in cardiovascular pathology. Front Physiol.

[54] Purcell BP, Barlow SC, Perreault PE (2018). Delivery of a matrix metalloproteinaseresponsive hydrogel releasing TIMP-3 after myocardial infarction: effects on left ventricular remodeling. Am J Physiol Heart Circ Physiol.

[55] Alberts BM, Sacre SM, Bush PG, Mullen LM (2019). Engeineering of TIMP-3 as a LAP-fusion protein for targeting to sites of inflammation. J Cell Mol Med.

[56] Tian H, Huang ML, Liu KY (2012). Inhibiting matrix metalloproteinase by cell-based TIMP-3 gene transfer effectively treats acute and chronic ischemic cardiomyopathy. Cell Transplant.

[57] Murphy G, Houbrechts A, Cockett MI, Williamson RA, O'Shea M, Docherty AJ (1991). The N-terminal domain of tissue inhibitor of metalloproteinases retains metalloproteinase inhibitory activity. Biochemistry.

[58] Geng Q, Sun X, Gong T, Zhang ZR (2012). Peptide-drug conjugate linked via a disulfide bond for kidney targeted drug delivery. Bioconjug Chem.

[59] Sun W, Li L, Li LJ, Yang QQ, Zhang ZR, Huang Y (2017). Two birds, one stone: dual targeting of the cancer cell surface and subcellular mitochondria by the galectin-3-binding peptide G3–C12. Acta Pharmacol Sin.

[60] Chen S-C, Kuo P-L (2016). The role of galectin-3 in the kidneys. Int J Mol Sci.

[61] Rubin JD, Nguyen TV, Allen KL, Ayasoufi K, Barry MA (2019). Comparison of gene delivery to the kidney by adenovirus, adeno-associated virus, and lentiviral vectors after intravenous and direct kidney injections. Hum Gene Ther.

[62] Wang J, Gao Y, Ma M (2013). Effect of miR-21 on renal fibrosis by regulating MMP-9 and TIMP1 in kk-ay diabetic nephropathy mice. Cell Biochem Biophys.

[63] Kölling M, Kaucsar T, Schauerte C (2017). Therapeutic miR-21 silencing ameliorates diabetic kidney disease in mice. Mol Ther.

[64] Roy D, Modi A, Khokhar M (2021). Microrna 21 emerging role in diabetic complications: a critical update. Curr Diabetes Rev.

[65] Hua F (2020). New insights into diabetes mellitus and its complications: a narrative review. Ann Transl Med.

[66] Darshi M, Van Espen B, Sharma K (2016). Metabolomics in diabetic kidney disease: unraveling the biochemistry of a silent killer. Am J Nephrol.

[67] Patti GJ, Yanes O, Siuzdak G (2012). Innovation: metabolomics: the apogee of the omics trilogy. Nat Rev Mol Cell Biol.

